# LOX-1, the Common Therapeutic Target in Hypercholesterolemia: A New Perspective of Antiatherosclerotic Action of Aegeline

**DOI:** 10.1155/2019/8285730

**Published:** 2019-11-30

**Authors:** Abhilasha Singh, Ashok Kumar Srinivasan, Lakshmi Narasimhan Chakrapani, Periandavan Kalaiselvi

**Affiliations:** ^1^Department of Medical Biochemistry, Dr. ALM Post Graduate Institute for Basic Medical Sciences, University of Madras, Chennai, India; ^2^Preclinical Stroke Modelling Laboratory, Burke Neurological Institute, Weill Cornell Medicine, White Plains, New York 10605, USA; ^3^Department of HIV, National Institute for Research in Tuberculosis, Chennai, India

## Abstract

**Background:**

Lectin-like oxidized low-density lipoprotein receptor-1 (LOX-1) is the major receptor for oxidized low-density lipoprotein (Ox-LDL) in the aorta of aged rats. Ox-LDL initiates LOX-1 activation in the endothelium of lipid-accumulating sites of both animal and human subjects of hypercholesterolemia. Targeting LOX-1 may provide a novel diagnostic strategy towards hypercholesterolemia and vascular diseases.

**Hypothesis:**

This study was planned to address whether aegeline (AG) could bind to LOX-1 with a higher affinity and modulate the uptake of Ox-LDL in hypercholesterolemia.

**Study Design:**

Thirty-six Wistar rats were divided into six groups. The pathology group rats were fed with high-cholesterol diet (HCD) for 45 days, and the treatment group rats were fed with HCD and aegeline/atorvastatin (AV) for the last 30 days. *In vivo* and *in vitro* experiments were carried out to assay the markers of atherosclerosis like Ox-LDL and LOX-1 levels. Histopathological examination was performed. Oil Red O staining was carried out in the IC-21 cell line. Docking studies were performed.

**Results:**

AG administration effectively brought down the lipid levels induced by HCD. The lowered levels of Ox-LDL and LOX-1 in AG-administered rats deem it to be a potent antihypercholesterolemic agent. Compared to AV, AG had a pronounced effect in downregulating the expression of lipids evidenced by Oil Red O staining. AG binds with LOX-1 at a higher affinity validated by docking.

**Conclusion:**

This study validates AG to be an effective stratagem in bringing down the lipid stress induced by HCD and can be deemed as an antihypercholesterolemic agent.

## 1. Introduction

Atherosclerosis is a pathological condition characterized by lipid infiltration and plaque formation in the arteries. There are numerous risk factors associated with atherosclerosis: aging and age-associated changes in gene expression of the arterial wall have been proposed among the most important risk factors [1]. Age-associated arterial changes may contribute to the pathological events in atherosclerosis like hyperplasia, medial thickening, endothelial dysfunction that augments monocyte/endothelial adherence, enhanced endothelial cell apoptosis, and diminished vascular cell replicative capacity [2]. Hyperlipidaemia and reactive oxygen species formation (ROS) are the other important factors in the initiation and progression of atherosclerosis [3]. Low-density lipoprotein (LDL) cholesterol is an established risk factor for coronary artery disease: in the presence of oxidative stress, these LDL particles get oxidized to form a lipoprotein species that is particularly atherogenic in nature. These oxidized-LDL contributes to the atherosclerotic plaque initiation and progression through a myriad of mechanisms which includes the induction of endothelial cell activation/dysfunction, macrophage-induced foam cell formation, and smooth muscle cell migration and proliferation [4]. The biological effects of Ox-LDL are mediated via a number of molecules such as scavenger receptor SR AI/II, SR B1, CD36, and LOX-1 [5]. Li et al. [6] are the pioneers in identifying that lectin-like oxidized low-density lipoprotein receptor 1 (LOX-1) is the critical molecule that is responsible for Ox-LDL uptake by endothelial cells. LOX-1 is a type II membrane protein comprising of four domains, and the c-terminal end residues and several conserved positively charged residues spanning the lectin domain are essential for Ox-LDL binding. Besides Ox-LDL, LOX-1 can recognize apoptotic/aged cells, activated platelets, and bacteria, implying versatile physiological functions [7]. The major contribution of LOX-1 to the atherogenic events has been confirmed in animal models. LOX-1 knockout mice exhibit reduced intimal thickness and inflammation and increased expression of protective factors [8]. On the contrary, LOX-1-overexpressing mice present an accelerated atherosclerotic lesion formation which is associated with increased inflammation [9]. LOX-1 activation by Ox-LDL causes endothelial changes that are characterized by the activation of nuclear factor-*κ*B through an increased reactive oxygen species, subsequent induction of adhesion molecules, and endothelial apoptosis [10]. Taken together, these findings support the possible contribution of LOX-1 in the pathogenesis of atherosclerosis, and identification of antagonists for LOX-1 might be a good therapeutic approach to vascular diseases.

Statins (3-hydroxy-3-methylglutaryl coenzyme A reductase inhibitors) are the first-line choice for lowering total and LDL cholesterol levels and they have been proven to reduce the risk of coronary artery disease. Recent data suggest that these compounds, in addition to their lipid-lowering ability, can also reduce the production of reactive oxygen species and increase the resistance of LDL to oxidation [11]. Clinical evidence also suggests that atorvastatin has been found to reduce the risk of cardiovascular disease by decreasing serum total cholesterol and low-density lipoprotein cholesterol in coronary artery diseases [12]. Studies by Biocca et al. [13] suggested the interaction of atorvastatin to LOX-1. Accordingly, atorvastatin has been selected to be the standard drug in the present study. Even though statins are the established treatment for hypercholesterolemia and cardiovascular diseases, they are associated with skeletal muscle, metabolic, neurological, and other possible side effects. This persuades us to look for an alternative therapy with lesser side effects and one that has a phytomedicine base [14]. Aegeline is a purified compound obtained from the leaf extract of the plant “*Aegle marmelos*.” The plant has been explored extensively for the presence of bioactive compounds and for its therapeutics. However, aegeline is one among the compounds comparatively less explored for its potential in targeting LOX-1 and this study is among the first studies designed to explore that potential. Aegeline shows a structural similarity to that of beta 3 adrenergic receptor agonists [15] which have been found to have a significant role in lipolysis, which is why the current study was designed to harness this potential of aegeline.

## 2. Materials and Methods

### 2.1. Source of Chemicals

Aegeline was procured from Clearsynth Canada. Bovine serum albumin (BSA) and the primers for genes of interest were procured from Sigma-Aldrich, USA. An iScript cDNA synthesis kit was purchased from Bio-Rad USA, and an enhanced chemiluminescence (ECL) kit was purchased from Millipore Corporation, USA. RPMI (Roswell Park Memorial Institute) 1640 medium with ATCC modification (2 mM L-glutamine, 10 mM HEPES, 1 mM sodium pyruvate, 4500 mg/l glucose, and 1500 mg/l sodium bicarbonate for use in incubators using 5% CO_2_ in air), antibiotics, and fetal bovine serum were purchased from Invitrogen (Groningen, The Netherlands). Oil Red O was purchased from HiMedia Laboratories Pvt. Ltd. (India). All other chemicals used were of analytical grade and were obtained from Medox Biotech, India Pvt. Ltd. (Sisco Research Laboratories Pvt. Ltd. (SRL)), Genei Laboratories Pvt. Ltd., and CDH (Central Drug House Pvt. Ltd., Mumbai, India).

### 2.2. Animals

Male albino rats of wistar strain were obtained from the Central Animal House Facility, University of Madras, Taramani Campus, and experiments were conducted in accordance with guidelines approved by the Institutional Animal Ethical Committee (IAEC No. 01/19/2014). The animals were housed two per cage in large spacious cages under conditions of controlled temperature (25.2°C) with 12/12 h light/dark cycle and were given food and water *ad libitum*.

### 2.3. Study Design

The animals were divided into the following six groups with six animals in each group:

1. Group 1—control (3-month-old) rats fed with normal rat feed for 45 days
2. Group 2—aged-control (24-month-old) rats fed with normal rat feed for 45 days
3. Group 3—aged rats fed with HCD for 45 days
4. Group 4—aged rats fed with HCD for 45 days and supplemented with AG (20 mg/kg of body weight) for the last 30 days [16]
5. Group 5—aged rats fed with HCD for 45 days and supplemented with AV (1.5 mg/kg of body weight) for the last 30 days [17]
6. Group 6—aged rats fed with a normal diet and administered AG alone for the last 30 days (hypercholesterolemia was induced by HCD comprising normal rat chow supplemented with 4% cholesterol, 1% cholic acid, and 30% coconut oil [16])

At the end of the experimental period, rats were anesthetized with ketamine (22 mg/kg b.wt., i/p) and aorta were excised immediately, immersed in ice-cold physiological saline, and weighed. A 10% tissue homogenate was prepared by using Tris-HCl buffer (0.01 M) pH 7.4 for biochemical assays. The rest of the tissue was stored at -80°C for protein expression studies. Blood samples were collected by cardiac puncture into anticoagulant-containing and anticoagulant-free test tubes. Blood samples were kept at room temperature for 30 min, allowed to clot, and then centrifuged at 3000 rpm for 10 min to collect the serum. Plasma was collected by the centrifugation of the anticoagulated blood. Small sections from each tissue were kept aside for histological studies. Part of the tissue was fixed in formalin (10% formaldehyde) for immunohistochemical analysis. Paraffin wax sections were prepared with the fixed tissues.

### 2.4. Lipid and Lipoprotein Profile

Total cholesterol (TC), triglycerides (TG), and high-density lipoproteins (HDL) in serum were assessed using commercial kits that spin react in a semiautomatic analyzer (RX Monza, Randox, UK).

#### 2.4.1. LDL (Low-Density Lipoprotein) and VLDL (Very Low-Density Lipoprotein)

LDL cholesterol was calculated according to the Friedwald formula:

(1) $$\begin{array}{c} \text{LDL}=\text{total cholesterol}-\left(\text{VLDL}+\text{HDL cholesterol}\right),\\ \text{VLDL }\left(\text{very low}‐\text{density lipoprotein}\right)=\frac{\text{TG}}{5}. \end{array}$$

The values of total cholesterol, triglycerides, HDL, VLDL, and LDL are expressed as mg/dl.

#### 2.4.2. Atherogenic Index

AI was calculated as follows:

(2) $$\begin{array}{c} \text{AI}=\log\left(\frac{\text{TG}}{\text{HDL}‐\text{C}}\right). \end{array}$$

#### 2.4.3. Free Fatty Acids

Tissue free fatty acids were estimated by the method of Hron and Menahan [18]. Free fatty acid content in tissue is expressed as *μ*E/l.

### 2.5. Histopathological Studies

The histology of aorta was studied using haematoxylin and eosin (H&E) staining. A portion of the aorta tissue was fixed in 10% buffered formalin. The washed tissues were dehydrated in the descending grades of isopropanol and finally cleared in xylene. The tissues were then embedded in molten paraffin wax. Sections were cut at 5 *μ*m thickness and stained with haematoxylin and eosin. The sections were then viewed under a light microscope (Nikon “ECLIPSE E400” microscope, Japan) for histopathological changes.

### 2.6. Assay of Oxidized LDL by ELISA

The levels of Ox-LDL in serum were measured using ELISA as described by Crowther [19]. The levels are expressed as pg/ml.

### 2.7. Western Blot Analysis

The tissue homogenate was prepared in 50-100 *μ*l of lysis buffer (with protease inhibitors) and centrifuged, and the supernatant was collected. Total tissue extracts containing 50-100 *μ*g of protein samples were prepared in sodium dodecyl sulphate (SDS) sample buffer (Sigma-Aldrich) and were separated by SDS-electrophoresis on 10%-12% polyacrylamide gels, further transferred to a polyvinylidene difluoride (PVDF) membrane prior to immune detection, and subjected to western blot analysis. The antibodies against LOX-1 (goat anti-rabbit IgG-HRP conjugate, 1 : 1000) were purchased from Abcam and were used to detect protein levels in the aorta tissues. To verify the uniformity of the protein load and transfer efficiency across the test samples, membranes were reprobed with beta-actin (Cell Signalling Technology, 1 : 1000 dilution). Immunoreactive bands were developed by Immobilon Western Chemiluminescent HRP Substrate (Millipore Corporation, Billerica, USA), visualized using an enhanced chemiluminescence system (ChemiDoc, Bio-Rad, USA), and presented in comparison to beta-actin expression.

### 2.8. Cell Culture

Monolayer cultures of IC-21 cells (NCCS, Pune, India) were cultured in RPMI 1640 with ATCC modification supplemented with 10% heat-inactivated fetal bovine serum (FBS) and antibiotics (mixture 1% penicillin/streptomycin/nystatin). Cells were incubated in T25 tissue culture flasks at 37°C in a humidified atmosphere (5% CO_2_ and 95% air environment). Aegeline was dissolved in 0.1% DMSO, and Ox-LDL was suspended in PBS. Aegeline at a concentration of 10 *μ*M (based on a dose-fixation study) was used for the studies. The cells were then divided into five groups for various experiments to be performed (Group 1—IC-21 cells were maintained in RPMI 1640 without any treatment; Group 2—IC-21 cells were exposed to Ox-LDL at a concentration of 100 *μ*g/ml for 24 hours; Group 3—IC-21 cells were treated with aegeline followed by Ox-LDL at a concentration of 100 *μ*g/ml for 24 hours; Group 5—IC-21 cells were treated with atorvastatin (20 *μ*g) [20] followed by Ox-LDL at a concentration of 100 *μ*g/ml [21] for 24 hours; and Group 5—IC-21 cells were treated with aegeline alone.

### 2.9. Oil Red O Staining

Oil Red O staining was performed as prescribed by Lillie and Ashburn [22]. Cells were plated in 24-well tissue culture plates (Costar) at a density of 1 × 10^5^ cells/ml and upon reaching subconfluence, the macrophages were incubated with Ox-LDL (100 *μ*g/ml) for 24 h, the medium was aspirated, and the cells were rinsed twice with 0.01 M PBS. Cells were fixed with 4% PFA for 10 min and then rinsed in PBS for 3 times. To this, 60% isopropanol was added and incubated for 5 min and rinsed. The working solution of Oil Red O stain was added and set aside for 5 min, then washed with PBS. Haematoxylin was added and allowed to stand for 1 min, and tissues were washed with PBS to remove residual stain. The slides were viewed under phase contrast microscope; lipids appeared red and the nuclei appeared blue.

### 2.10. In Silico Study

Geometry-optimized molecular structures for aegeline and atorvastatin were obtained using the iGEMDOCK (a generic evolutionary method for molecular docking) automated docking program. iGEMDOCK is a software used for integrated structure-based virtual screening, molecular docking, postscreening analysis, and visualization step. The 3-dimensional (3D) coordinates of four target proteins were selected and obtained from the protein data bank (PDB). The PDB id of LOX-1 (1YPQ) was selected for the in silico study. The 3D structure coordinates of each therapeutic target protein and ligand molecules were implemented through the GEMDOCK-graphical environment interface. Before doing docking analysis, the output path was set. GEMDOCK default parameters included the population size (*n* = 200), generation (*g* = 70), and number of solutions (*s* = 10) to compute the probable ligand-binding mechanism for each target protein LOX-1. Then the docking run was started using a GEMDOCK scoring function. After docking, the individual binding pose of each ligand was observed and their binding affinity with the target proteins were analyzed. Visual examination of the predicted binding geometries (docking poses) thereby contributes crucially to the further development of a lead compound. In the postdocking screening, the best-binding pose and total energy of each ligand were analyzed. The details of the best-binding pose and total energy values were saved in an output folder. The protein-ligand binding site was analyzed and visualized by using PyMOL.

### 2.11. Statistical Analysis

Data are presented as mean ± standard error of mean (SEM) of the results obtained from the average of at least three to six independent experiments. Results were analyzed by one-way analysis of variance (ANOVA) using the SPSS software package for Windows (version 20.0; SPSS Inc., Chicago, IL, USA) and *p* values were determined using the Student-Newman-Keuls and least significant difference post hoc tests. Differences among means were considered statistically significant when the *p* value was less than 0.05.

## 3. Results (In Vivo)

### 3.1. Serum Lipid and Lipoprotein Profile

Table 1 shows the impact of aegeline on the serum lipid profile of experimental groups. Assessment of the serum lipid profile in the present study reveals that there was a significant (*p* < 0.05) increase in the levels of serum total cholesterol (1.37-fold), triglycerides (1.21-fold), LDL (1.55-fold), VLDL (1.22-fold), and free fatty acids (FFA) (1.15-fold) along with a concomitant decrease in HDL (1.57-fold) in HCD-alone-fed aged rats when compared to the aged control rats. Aegeline and atorvastatin supplementation to HCD-fed rats demonstrated a significant decline (*p* < 0.05) in serum total cholesterol, triglycerides, LDL, VLDL, and FFA with a concomitant increase (*p* < 0.05) in HDL, when compared to the HCD-alone-fed group. Moreover, it was observed that aegeline and atorvastatin have almost similar beneficial effects on reducing lipid levels in the serum. The atherogenic index was found to reach the maximum in the HCD-fed groups as expected, and aegeline and atorvastatin were capable of bringing down the atherogenic index to normal levels.

### 3.2. Histopathological Studies

**Figure 1:**
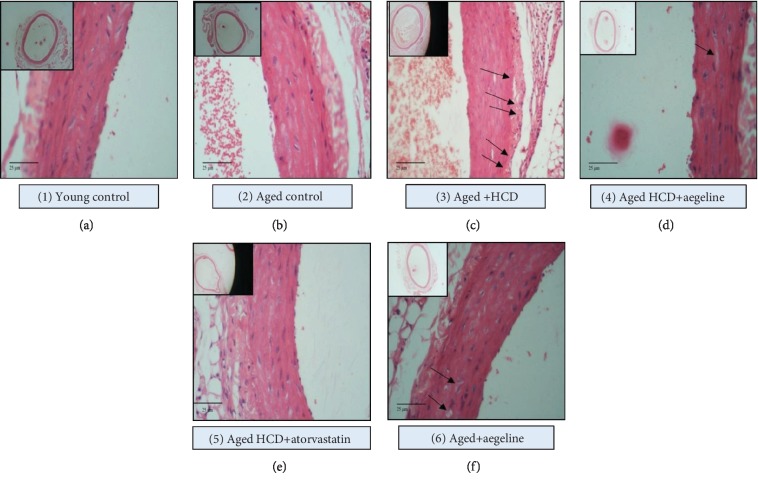
Histopathological sections of aorta in various experimental groups fed with HCD and the efficacy of aegeline (20x). Light microscopic analysis of the pathological H&E-stained aorta sections of rats (20x). (a) Young control (Group 1): aorta shows normal tissue architecture such as even thickness and inner and outer elastic plates with no signs of lipid deposition or necrosis. (b) Aged control (Group 2): aorta shows lean deterioration in the tissue architecture with considerable increase in tissue thickness. (c) Aged+HCD (Group 3): aorta shows necrotic core in tunica media which could possibly mean lipid deposition and cell vacuolization. (d) Aged+HCD+aegeline (Group 4): aorta shows no signs of abnormal architecture in the tunica intima and medial layers; however, there is mild cell vacuolization. (e) Aged+HCD+atorvastatin (Group 5): aorta shows lean deterioration in the tissue architecture and cell vacuolization. (f) Aged+aegeline (Group 6): aorta shows thin and smooth vessel wall with even thickness.

**Figure 2:**
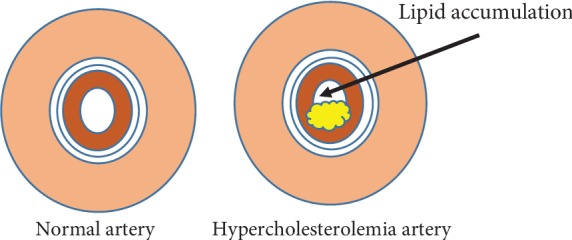
The pictorial representation of normal and HCD-challenged artery.

The haematoxylin and eosin staining in the aorta sections of rats is shown in Figure 1. The young control group showed normal tissue architecture, such as even thickness and inner and outer elastic plates with no signs of lipid deposition or necrosis. The aged control group had shown deterioration in tissue architecture with a considerable increase in tissue thickness, whereas the HCD-fed group showed a necrotic core in tunica media which could possibly mean lipid deposition and cell vacuolization. The illustration in Figure 2 is a pictorial representation showing the lipid accumulation in the HCD artery. Aegeline treatment to HCD-challenged rats showed no signs of abnormal architecture in the tunica intimal and medial layers. Though there were small signs of cellular vacuolization in atorvastatin-treated rats, there was no increase in thickness of the intimal layer as observed in the microscopic image. The aegeline-alone-treated aorta of aged rats showed no changes in the aortic architecture.

### 3.3. Assay of Ox-LDL by ELISA

**Figure 3:**
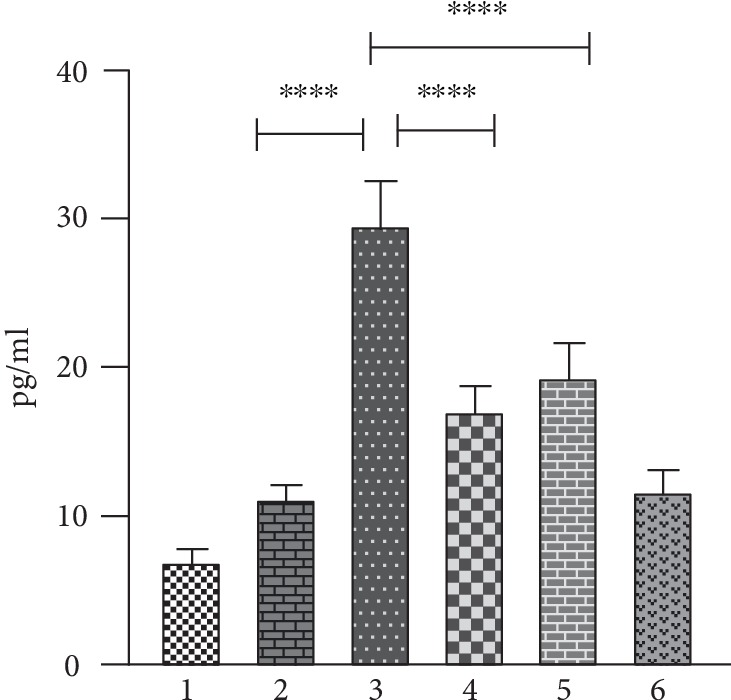
Aegeline mitigates the HCD-induced increase in the levels of serum Ox-LDL in aged experimental rats. Each bar represents mean ± SEM for six rats in each group. Values are statistically significant at the level of *p* < 0.05, where Group 2 is compared with Group 1 and Group 3 is compared with Group 2. Groups 4 and 5 are compared with Group 3, and Group 6 is compared with Group 2. Group 1: young control; Group 2: aged control; Group 3: aged+HCD; Group 4: aged+HCD+aegeline; Group 5: aged+HCD+atorvastatin; Group 6: aged+aegeline.

Figure 3 displays the levels of Ox-LDL in the serum of various experimental groups. The assessment of the serum levels of Ox-LDL in HCD-fed rats revealed that there was a significant increase (2.66-fold) when compared to that of aged control rats. On the other hand, supplementation of aegeline/atorvastatin to HCD-fed rats brought down the serum levels of Ox-LDL when compared to HCD-alone-fed rats by 1.52- and 1.72-fold, respectively.

### 3.4. AG and AV: Effect on the Protein Expression Profile of LOX-1

**Figure 4:**
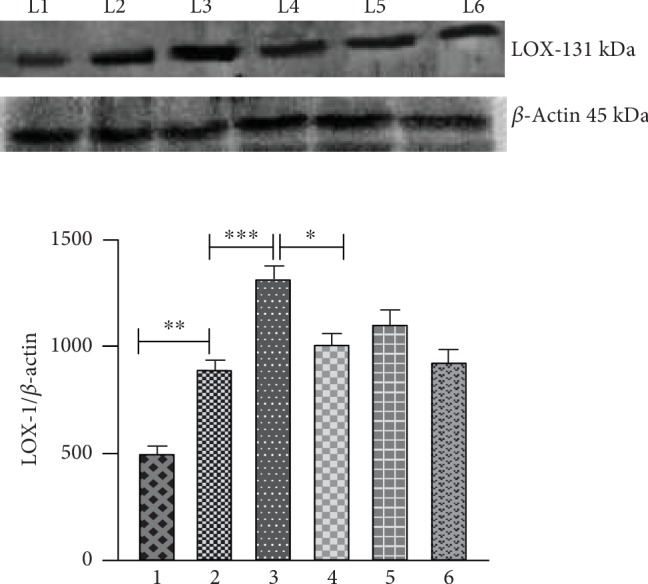
Effect of aegeline on the expression of scavenger receptors in the aorta of aged rats fed with HCD. Each bar represents mean ± SEM for six rats in each group. Values are statistically significant at the level of *p* < 0.05, where Group 2 is compared with Group 1 and Group 3 is compared with Group 2. Groups 4 and 5 are compared with Group 3, and Group 6 is compared with Group 2. Group 1: young control; Group 2: aged control; Group 3: aged+HCD; Group 4: aged+HCD+aegeline; Group 5: aged+HCD+atorvastatin; Group 6: aged+aegeline.

The levels of scavenger receptor LOX-1 in the aorta of different experimental animals are depicted in Figure 4. LOX-1 levels were increased by 58.73% and 161.30% in aged control and HCD-fed rats when compared to the young rats. Drug treatments significantly brought down the levels comparable to that of the aged control rats with a greater percentage reduction observed with aegeline (31.05%) than atorvastatin (22.70%).

### 3.5. In Vitro

#### 3.5.1. Oil Red O Staining

**Figure 5:**
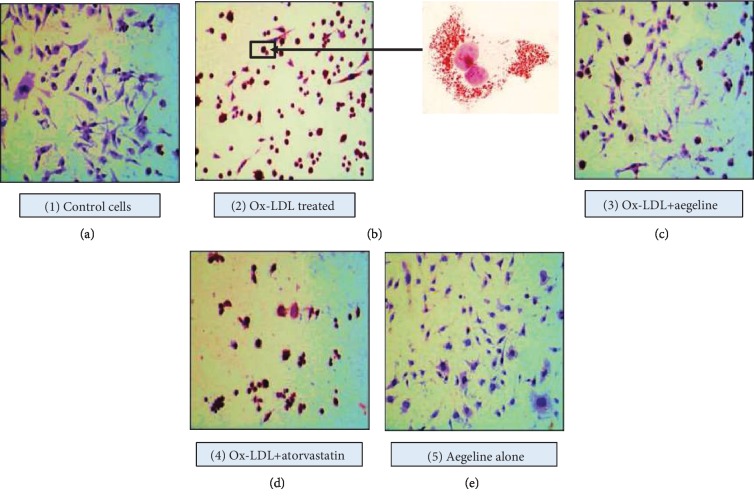
Oil Red O lipid staining in IC-21 cells treated with Ox-LDL and the lipid-lowering effect of aegeline.

Figure 5 represents the Oil Red O staining of IC-21 cells, where Group 1 control cells showed normal nuclear morphology with no signs of lipid staining in the cytoplasm of cells. In cells incubated with Ox-LDL (Group 2), there was an observed red staining in the cytoplasm and around the nucleus, which is a positive sign of foam cell formation. On the other hand, aegeline (Group 3) showed less Oil Red O staining in the cytoplasm. However, atorvastatin treatment showed Oil Red O uptake with lowered significance as compared to aegeline. Aegeline-alone-treated cells showed normal cellular morphology.

### 3.6. Result of LOX-1 Docking Studies

#### 3.6.1. Results of In Silico Studies

**Figure 6:**
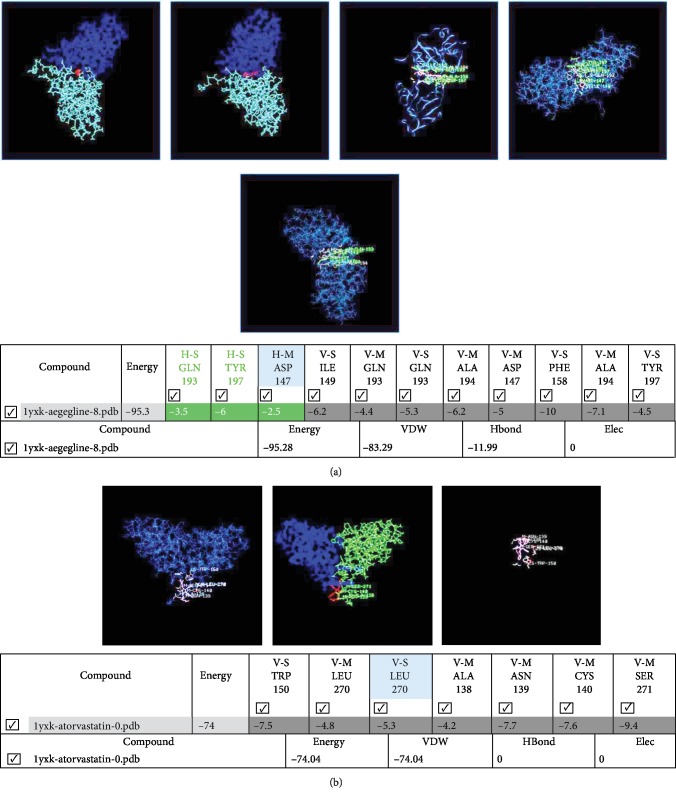
(a) Molecular docking of LOX-1 with aegeline. (b) Molecular docking of LOX-1 with atorvastatin.

Docking studies have been carried out by taking 1VKX to understand the nature of the interaction of aegeline and atorvastatin with LOX-1. Performing a stable docking procedure in iGEMDOCK by taking LOX-1 as a target with aegeline resulted in the most stable drug receptor complex with a LOX-1-aegeline docking score of -95.28 kcal/mol (Figure 6(a)) and an atorvastatin docking score of -74.04 kcal/mol (Figure 6(b)). The docking of aegeline with oxidized-LDL receptors exhibited a strong interaction, and the results showed that aegeline has extensive interactions with Ile-149, Gln-193, Ala-194, Asp-147, Phe-158, and Try-197 of one subunit and Gln-193, Tyr-197, and Asp-147 of another subunit of a LOX-1 homodimer with a fitness score of -95.3 kcal/mol.

## 4. Discussion

### 4.1. Effect of Aegeline on HCD-Induced Lipid Abnormalities

Being overweight or obese is associated with alterations in plasma lipids and a broad spectrum of cardiometabolic disorders, including an increase in plasma low-density lipoprotein cholesterol (LDL-C) and triglyceride (TG) concentrations [16, 23]. On analysis of the lipid profile, we found that total cholesterol and LDL cholesterol were significantly high in the aged group when compared to young control rats. Elevated plasma LDL-C levels represent one of the key causal factors for the development of atherosclerosis and subsequent coronary artery disease [4]. In addition, the studies have also found a gradual decline in the fractional clearance of LDL from the circulation with age and reduced expression of hepatic LDLRs with increasing age in some species [16, 24]. In the present study, rats fed with HCD exhibited high levels of LDL, VLDL, triglycerides, total cholesterol, and free fatty acids (FFA) in serum as compared to aged rats fed with a normal diet. Similar alterations in lipid and lipoprotein parameters in rats fed with HCD have been reported in earlier studies [16, 23] and the observed increase in the cholesterol levels in HCD-fed rats may be due to the presence of cholic acid in the diet, which might have been a responsible factor for the increased absorption of cholesterol in the intestine [16]. The supplementation of a high-cholesterol diet also reduced the HDL cholesterol levels, which is in corroboration with the available literature that state that increased levels of atherogenic lipids with a concomitant decrease in HDL is a characteristic feature observed in high-cholesterol-diet-fed rats [16]. The observed increased levels of cholesterol, LDL, and VLDL are also reflected in the atherogenic index calculated in the current study. The hypocholesterolemic activity of atorvastatin, a statin by origin, has been attributed to its multifaceted efficacy in modulating cholesterol metabolism. Moreover, Liu et al. [17] have demonstrated that the hypocholesterolemic activity of atorvastatin is mainly attributed to its ability to inhibit or decrease the production of apoB-containing lipoproteins. Not only aegeline as observed in the current study but even its other derivatives obtained from the *Aegle marmelos* plant have also been shown to have antihyperlipidemic activities [15]. The leaf extract of *Aegle marmelos* is shown to exert an antihyperlipidemic potential in HCD rats, where there is altered lipid metabolism [22]. Aegeline has also been shown to possess both anti-inflammatory and antihyperlipidemic activity as observed in our previous study in fatty liver conditions [16]. Hence, both aegeline and atorvastatin decrease the atherogenic risk, thereby affording cardioprotection. Moreover, the efficacy of aegeline in combating hypercholesterolemia is more or less similar to that of atorvastatin.

### 4.2. Effect of Aegeline on HCD-Induced Atherosclerotic Plaque Progression

In concordance with the above observations on lipid anomalies in HCD-fed rats, the histological findings in this study on the aortic wall demonstrated that there was an increase in the tunica medial thickness most probably due to lipid deposition in the aorta. The thickening of the aortic intima is one of the key features of atherosclerosis and correlated well with the biochemical parameters observed in atherogenic-diet-fed rats. Zhou et al. [25] have also reported that HCD feeding to rats results in a marked thickening of the intima. Carotid intima thickness measurements have been commonly used in observational studies as a marker for atherosclerosis to evaluate its determinants and consequences in various populations [26]. These effects were abrogated with the administration of atorvastatin for 30 days. A study done by Taylor et al. [27] has stated that atorvastatin had a significant impact on the reduction of intima-media thickness. Another follow-up study has shown that statin drugs including atorvastatin were seen to reduce carotid intima-media thickness along with soft plaque regression [28]. On aegeline supplementation, the intima was found to be near normal.

Both atorvastatin and aegeline have shown anti-inflammatory action [16], and so it might improve endothelial function. An improvement in endothelial function would have resulted in a reduction of intimal thickness.

### 4.3. Effect of Aegeline on Serum Ox-LDL

When oxidative stress is present, ROS may modify or damage lipids, proteins, and DNA with deleterious consequences for vascular function and structure [29]. LDL is one such lipoprotein more susceptible to oxidative modifications, and several studies performed over the past decade illustrate that the oxidatively modified form of LDL is more accountable than native LDL in the progression of atherogenesis [4]. Our studies also have identified Ox-LDL levels to be high in the serum of HCD-fed rats which is in concurrence with the available literature [5]. According to the classical hypothesis, Ox-LDL accumulates in the atherosclerotic lesions over a prolonged period of time. But recent studies carried out with aged rats show changes in the level of Ox-LDL, and this signifies the dynamics of the Ox-LDL state that it can equilibrate between circulation and tissues [30]. Hence, the increased Ox-LDL levels in the plasma of HCD-fed rats is the true reflection of the pathogenic process that occurs in the rat aorta. Retention of LDL in the vessel wall with subsequent oxidation is also considered to be an important event in the early stages of an atherosclerotic lesion playing a key role in endothelial dysfunction and atherogenesis. Oxidized LDL, in fact, activates endothelial cells by inducing the expression of several cell surface adhesion molecules which mediate the rolling and adhesion of blood leukocytes (monocytes and T cells); after adhesion to the endothelium, leukocytes migrate into the intima in response to chemokines. Monocytes then differentiate into macrophages that upregulate both Toll-like receptors (TLRs) and scavenger receptors (SRs) that internalize Ox-LDL, leading to lipid accumulation and foam cell formation [5]. Therefore, increased levels of Ox-LDL might be responsible for initiating the events involved in plaque formation as substantiated by intimal thickness in the HCD-fed animal aortas. Aegeline or atorvastatin supplementation has brought down the serum levels of Ox-LDL in rats challenged with HCD. In vitro studies conducted by Sarkar et al. [31] have identified that structural analogs of aegeline are potent inhibitors of LDL oxidation. However, so far no reports have been stated with reference to the impact of aegeline on serum levels of oxidized LDL. On the other hand, Fuhrman et al. [32] have reported that atorvastatin therapy decreases serum lipid and Ox-LDL levels. It is obvious from our observations that aegeline mitigates cholesterol-mediated ROS to a greater extent by reducing the levels of cholesterol by its antihypercholesterolemic effect indirectly, since it was able to reduce the levels of Ox- LDL.

### 4.4. Effect of Aegeline on Receptors of Ox-LDL

The mechanism of Ox-LDL uptake is widely studied. Early work suggested that the uptake of oxidized LDL occurs via a scavenger receptor, the lectin-like oxidized low-density lipoprotein receptor-1 (LOX-1) [5]. LOX-1 has been identified first in endothelial cells as the major Ox-LDL receptor; however, also macrophages and smooth muscle cells express LOX-1 together with other scavenger receptors, altogether contributing to the induction of endothelial dysfunction by several mechanisms [5]. The potential role of LOX-1 in the pathogenesis of atherosclerosis includes endocytosis of Ox-LDL, expression colocation with atherosclerosis enhanced by risk factors of atherosclerosis. The present study has demonstrated the enhanced manifestation in the levels of LOX-1 in aged and aged HCD rats concomitant with the previous studies which state that there is upregulated LOX-1 expression in the endothelium of HCD-fed rabbits [33]. Macrophages bind and internalize Ox-LDL through CD36, and thus activated macrophages secrete oxidants, including myeloperoxidase, which oxidizes LDL, and thus enlarges the pool of Ox-LDL [5]. Our studies have shown that both atorvastatin and aegeline are able to reduce the expression of LOX-1 in aged and aged HCD-fed rats.

### 4.5. Aegeline Augments Foam Cell Formation Induced by Ox-LDL

Macrophage-derived foam cell formation is the early hallmark of atherogenesis. Hence, we wanted to study the effect of aegeline and atorvastatin on foam cell formation in IC-21 macrophage cells. Oil Red O staining of IC-21 cells showed normal nuclear morphology with no signs of lipid staining in the cytoplasm. However, when cells were incubated with Ox-LDL, they showed the positive staining of Oil Red O inside the cytosol due to the uptake of Ox-LDL inside these cells. This is in corroboration with the previous study conducted by Yang et al. [33] stating that incubating macrophage cells with Ox-LDL resulted in extensive lipid accumulation in the cytosol. Aegeline/atorvastatin treatment has greatly reduced lipid accumulation in macrophage cells. These observations explicitly show that aegeline not only reduces Ox-LDL formation but also regulates the uptake of the same. One mechanism by which it can do this is by downregulating the scavenger receptor, for which data from in vivo studies support its downregulation (Figure 4). On the other hand, it might prevent the interaction between LOX-1 and Ox-LDL by binding to LOX-1. Hence, molecular docking studies were carried out using iGEMDOCK to find out if there is any interaction between LOX-1 and aegeline. LOX-1 is a protein that is made up of 273 residues comprising four domains. The first 36 residues form the cytoplasmic tail followed by a single transmembrane domain made up of amino acids from 37 to 58 and an extracellular region containing two domains, the first one comprising residues from 58 to 142 and the second from 143 to 273 which is a C-type lectin-like domain (CTLD) responsible for Ox-LDL recognition. Aegeline specifically has interaction with the CTLD domain. Francone et al. [34] have reported that mutations of certain residues present in the tunnel, particularly Ile-149, impair binding to Ox-LDL, confirming the crucial role of the tunnel in ligand interaction and binding. Furthermore, Biocca et al. [13] have docked atorvastatin with LOX-1 and found that Ile-149 of LOX-1 interacts with statins and Ile-149 is always involved in the stability of contact with these drugs. Point mutations on Ile-149, which point to the empty space in the center of the tunnel, markedly reduce the binding of Ox-LDL and a series of oxidized phospholipids. The “clamp motion” mechanism, which is hypothesized as a mechanism for Ox-LDL catching, is only observed in the absence of ligands and blocked when the CTLD hydrophobic tunnel is filled. Docking results showed that aegeline has extensive interaction with Ile-149, Gln-193, Ala-194, Asp-147, Phe-158, and Try-197 of one subunit and Gln-193, Tyr-197, and Asp-147 of another subunit of the LOX-1 homodimer with a fitness score of -95.3. Aegeline also binds to Ile-149 indicating a stable interaction. Docking atorvastatin with LOX-1 has docking energy of -74.04 which is slightly lower than that of aegeline with LOX-1, thereby showing more favorable binding than the statin drug. Hence, LOX-1 seems to be an attractive target for the therapy of a number of cardiovascular diseases. In this regard, aegeline has a tremendous potential to inhibit LOX-1 which needs to be further explored.

## 5. Summary/Conclusion

Aegeline at a concentration of 20 mg/kg body weight is effective in reducing the lipid anomalies in aged hypercholesterolemic rats when compared to atorvastatin by targeting LOX-1 as observed in western blot and docking studies. Aegeline had a pronounced effect in downregulating the expression of lipids evidenced by oxidized-LDL expression and Oil Red O staining. This study validates aegeline as a potent antihypercholesterolemic agent.

## Figures and Tables

**Table 1:** Aegeline alters the HCD-induced lipid anomaly in aged hypercholesterolemic rats.

Parameters	Group 1	Group 2	Group 3	Group 4	Group 5	Group 6
TC (mg/dl)	132.10 ± 9.37	207.59 ± 15.65^a^	284.63 ± 21.34^ab^	215.27 ± 22.61^c^	220.44 ± 16.29^c^	181.07 ± 13.39^b^
VLDL (mg/dl)	19.02 ± 1.42	22.35 ± 1.71^a^	27.01 ± 2.33^ab^	24.35 ± 21.42^c^	25.17 ± 2.25^c^	20.14 ± 1.57^b^
LDL (mg/dl)	72.71 ± 5.68	152.13 ± 12.37^a^	235.84 ± 22.43^ab^	161.01 ± 14.46^c^	167.94 ± 5.58^c^	122.32 ± 10.98^b^
HDL (mg/dl)	40.37 ± 3.14	33.11 ± 3.01^a^	21.79 ± 2.15^ab^	29.91 ± 2.37^c^	27.33 ± 2.55^c^	37.27 ± 1.86^b^
TG (mg/dl)	95.12 ± 7.63	111.75 ± 9.52^a^	135.22 ± 14.81^ab^	121.73 ± 11.56^c^	125.88 ± 12.81^c^	100.81 ± 9.30^b^
FFA (*μ*E/l)	211.27 ± 15.84	279.81 ± 25.51^a^	322 ± 33.83^ab^	291.52 ± 29.21^c^	295.19 ± 23.61^c^	252.36 ± 24.19^b^
Atherogenic index	0.37	0.527	0.792	0.608	0.663	0.431

Atherogenic index was calculated according to the following formula:log(TG/HDL − C). Each value represents mean ± SEM for six rats in each group. Values are statistically significant at the level of *p* < 0.05. ^a^Group 2 compared with Group 1. ^ab^Group 3 compared with Group 1 and Group 2. ^c^Group 4 compared with Group 3. Group 5 compared with Group 3. Group 6 compared with Group 2. Group 1: young control; Group 2: aged control; Group 3: aged+HCD; Group 4: aged+HCD+aegeline; Group 5: aged+HCD+atorvastatin; Group 6: aged+aegeline.

## Data Availability

The data used to support the findings of this study are included within the article.

## Abbreviations

CAD: Coronary artery disease
CTLD: C-type lectin-like domain
CVD: Cardiovascular disease
ELISA: Enzyme-linked immunosorbent assay
HCD: High-cholesterol diet
HDL: High-density lipoprotein
H&E: Haematoxylin and eosin
IC-21: Infected cell passage number 21
LDL: Low-density lipoprotein
LOX: Oxidized low-density lipoprotein receptor
Ox-LDL: Oxidized low-density lipoprotein
PBS: Phosphate-buffered saline
RPMI: Roswell Park Memorial Institute
VLDL: Very low-density lipoprotein.

## References

[1] Wanner C., Amann K., Shoji T. (2016). The heart and vascular system in dialysis. *The Lancet*.

[2] Zhu W., Kim B. C., Wang M. (2018). TGF*β*1 reinforces arterial aging in the vascular smooth muscle cell through a long-range regulation of the cytoskeletal stiffness. *Scientific Reports*.

[3] Chistiakov D. A., Orekhov A. N., Bobryshev Y. V. (2015). Contribution of neovascularization and intraplaque haemorrhage to atherosclerotic plaque progression and instability. *Acta Physiologica*.

[4] Head T., Daunert S., Goldschmidt-Clermont P. J. (2017). The aging risk and atherosclerosis: a fresh look at arterial homeostasis. *Frontiers in Genetics*.

[5] Di Pietro N., Formoso G., Pandolfi A. (2016). Physiology and pathophysiology of oxLDL uptake by vascular wall cells in atherosclerosis. *Vascular Pharmacology*.

[6] Li C., Zhang J., Wu H. (2017). Lectin-like oxidized low-density lipoprotein receptor-1 facilitates metastasis of gastric cancer through driving epithelial-mesenchymal transition and PI3K/Akt/GSK3*β* activation. *Scientific Reports*.

[7] Xie X., Zhang L., Lin Y. (2017). Imiquimod induced ApoE-deficient mice might be a composite animal model for the study of psoriasis and dyslipideamia comorbidity. *Journal of Dermatological Science*.

[8] Ding Z., Liu S., Wang X. (2015). Cross-talk between LOX-1 and PCSK9 in vascular tissues. *Cardiovascular Research*.

[9] Xie W., Li L., Zhang M. (2016). MicroRNA-27 prevents atherosclerosis by suppressing lipoprotein lipase-induced lipid accumulation and inflammatory response in apolipoprotein E knockout mice. *PLoS One*.

[10] Ji K. T., Qian L., Nan J. L. (2015). Ox-LDL induces dysfunction of endothelial progenitor cells via activation of NF-*κ*B. *BioMed Research International*.

[11] Margaritis M., Sanna F., Antoniades C. (2017). Statins and oxidative stress in the cardiovascular system. *Current Pharmaceutical Design*.

[12] Wadhera R. K., Steen D. L., Khan I., Giugliano R. P., Foody J. M. (2016). A review of low-density lipoprotein cholesterol, treatment strategies, and its impact on cardiovascular disease morbidity and mortality. *Journal of Clinical Lipidology*.

[13] Biocca S., Iacovelli F., Matarazzo S. (2015). Molecular mechanism of statin-mediated LOX-1 inhibition. *Cell Cycle*.

[14] Thompson P. D., Panza G., Zaleski A., Taylor B. (2016). Statin-associated side effects. *Journal of the American College of Cardiology*.

[15] Rajan S., Satish S., Shankar K. (2018). Aegeline inspired synthesis of novel *β*3-AR agonist improves insulin sensitivity *in vitro* and *in vivo* models of insulin resistance. *Metabolism*.

[16] Singh A., Gowtham S., Chakrapani L. N. (2018). Aegeline vs Statin in the treatment of Hypercholesterolemia: A comprehensive study in rat model of liver steatosis. *Functional Foods in Health and Disease*.

[17] Liu M. W., Liu R., Wu H. Y. (2017). Atorvastatin has a protective effect in a mouse model of bronchial asthma through regulating tissue transglutaminase and triggering receptor expressed on myeloid cells-1 expression. *Experimental and Therapeutic Medicine*.

[18] Hron W. T., Menahan L. A. (1981). A sensitive method for the determination of free fatty acids in plasma. *Journal of Lipid Research*.

[19] Crowther J. R. (2000). *The ELISA Guidebook*.

[20] Xiao H., Zhang Q., Lin Y., Reddy B. S., Yang C. S. (2008). Combination of atorvastatin and celecoxib synergistically induces cell cycle arrest and apoptosis in colon cancer cells. *International Journal of Cancer*.

[21] Chen Y., Chen M., Wu Z., Zhao S. (2013). Ox-LDL induces ER stress and promotes the adipokines secretion in 3T3-L1 adipocytes. *PLoS One*.

[22] Lillie R. D., Ashburn L. L. (1943). Supersaturated solutions of fat stains in dilute isopropanol for demonstration of acute fatty degeneration not shown by Herxheimer’s technique. *Archives Pathology*.

[23] Singh A., Chakrapani L. N., Ashok Kumar S. (2017). Ethanolic extract of *Aegle marmelos* mediates its hypocholesterolemic effect by retarding circulatory oxidized LDL formation via 12/15 lipoxygenase pathway. *European Journal of Biomedical and Pharmaceutical Sciences*.

[24] Liu H. H., Li J. J. (2015). Aging and dyslipidemia: a review of potential mechanisms. *Ageing Research Reviews*.

[25] Zhou J. M., Wang H. M., Lv Y. Z., Wang Z. Z., Xiao W. (2018). Anti-atherosclerotic effect of Longxuetongluo capsule in high cholesterol diet induced atherosclerosis model rats. *Biomedicine & Pharmacotherapy*.

[26] Asghari G., Dehghan P., Mirmiran P. (2018). Insulin metabolism markers are predictors of subclinical atherosclerosis among overweight and obese children and adolescents. *BMC Pediatrics*.

[27] Taylor A. J., Kent S. M., Flaherty P. J., Coyle L. C., Markwood T. T., Vernalis M. N. (2002). ARBITER: Arterial Biology for the Investigation of the Treatment Effects of Reducing Cholesterol: a randomized trial comparing the effects of atorvastatin and pravastatin on carotid intima medial thickness. *Circulation*.

[28] De Groot E., Van Leuven S. I., Duivenvoorden R. (2008). Measurement of carotid intima–media thickness to assess progression and regression of atherosclerosis. *Nature Reviews Cardiology*.

[29] Menini S., Iacobini C., Ricci C., Fantauzzi C. B., Pugliese G. (2015). Protection from diabetes-induced atherosclerosis and renal disease by D-carnosine-octylester: effects of early vs late inhibition of advanced glycation end-products in Apoe-null mice. *Diabetologia*.

[30] Shih C. M., Lin F. Y., Yeh J. S. (2019). Dysfunctional high density lipoprotein failed to rescue the function of oxidized low density lipoprotein-treated endothelial progenitor cells: a novel index for the prediction of HDL functionality. *Translational Research*.

[31] Sarkar S., Sonkar R., Bhatia G., Tadigoppula N. (2014). Synthesis of new N-acryl-1-amino-2-phenylethanol and N-acyl-1-amino-3-aryloxypropanols and evaluation of their antihyperlipidemic, LDL-oxidation and antioxidant activity. *European Journal of Medicinal Chemistry*.

[32] Fuhrman B., Koren L., Volkova N., Keidar S., Hayek T., Aviram M. (2002). Atorvastatin therapy in hypercholesterolemic patients suppresses cellular uptake of oxidized-LDL by differentiating monocytes. *Atherosclerosis*.

[33] Yang X., Zhang J., Chen L. (2018). The role of UNC5b in ox-LDL inhibiting migration of RAW264.7 macrophages and the involvement of CCR7. *Biochemical and Biophysical Research Communications*.

[34] Francone O. L., Tu M., Royer L. J. (2009). The hydrophobic tunnel present in LOX-1 is essential for oxidized LDL recognition and binding. *Journal of Lipid Research*.

